# The COVID-19 Vaccination Rollout in Tanzania: The Role of Coordination in Its Success

**DOI:** 10.3390/vaccines13050484

**Published:** 2025-04-30

**Authors:** Fredrick Rwegerera, Mwendwa Mwenesi, Belinda J. Njiro, Florian Tinuga, Pricilla Kinyunyi, Mary Rose Giattas, Alice Christensen, Ntuli Kapologwe, Adam Meshack, Joseline Ishengoma, Sophia A. Kagoye, Mwinyi I. Msellem, Mwanahamisi Hassan Magwangwala, Fatma Mohammed Kabole, Daniel Ali, Chizoba Wonodi

**Affiliations:** 1 United States Agency for International Development (USAID), Dar es Salaam P.O. Box 9130, Tanzania; frwegerera@usaid.gov; 2 Ministry of Health, Dodoma P.O. Box 743, Tanzania; mwendwa18@yahoo.com (M.M.); floriantinuga@yahoo.com (F.T.); kpricillah@yahoo.com (P.K.);; 3 Department of Epidemiology and Biostatistics, School of Public Health and Social Sciences, Muhimbili University of Health and Allied Sciences (MUHAS), Dar es Salaam P.O. Box 65001, Tanzania; belindaj.njiro@gmail.com; 4 Jhpiego, Johns Hopkins University Affiliate, Baltimore, MD 21231, USA; maryrose.giattas@jhpiego.org (M.R.G.); alice.christensen@jhpiego.org (A.C.); meshack.adam@jhpiego.org (A.M.); 5 President’s Office Regional Administration and Local Government, Dodoma P.O. Box 1923, Tanzania; ishengomaj@yahoo.com (J.I.); mwanahamisi.magwangwala@tamisemi.go.tz (M.H.M.); 6 National Institute for Medical Research Mwanza Research Centre, Mwanza P.O. Box 1462, Tanzania; sophyadam90@gmail.com; 7 Ministry of Health, Zanzibar P.O. Box 236, Tanzania; mwinyi.msellem@yahoo.com (M.I.M.); fatumaepi@yahoo.com (F.M.K.); 8 International Vaccine Access Center, Department of International Health, Johns Hopkins Bloomberg School of Public Health, Johns Hopkins University, Baltimore, MD 21205, USA; dali2@jhmi.edu

**Keywords:** COVID-19 vaccination, vaccine rollout, multi-level coordination, coordination mechanisms, emergency management, Tanzania

## Abstract

**Background**: The national rollout of a vaccine is a complex and significant undertaking, made more challenging when the health system is experiencing shock, such as in a pandemic. Tanzania had relative success in its COVID-19 vaccination rollout compared to other African countries. **Objectives**: To better understand factors that contributed to this success, we examined the role of coordination (one of the six immunization system building blocks) on the outcomes of the national vaccine rollout. **Methods**: We obtained qualitative information from the published literature, COVID-19 vaccination program documents for Tanzania Mainland and Zanzibar, and reports from two documentation workshops with national, regional, and district stakeholders from the government, partners, academia, and civil society. Triangulating this information, we describe the COVID-19 vaccination coordination structure, the roles and responsibilities of its members, and the changes in their engagement and activities over the 18 months following the introduction of the COVID-19 vaccine. We also obtained quantitative data from the CHANJOCOVID system to analyze time trends in national COVID-19 vaccine coverage rates for the period August 2021 to December 2022. **Results**: We found that Tanzania had a multi-level, multi-partner integrated coordination mechanism that provided strategic direction, oversight, and guidance for the vaccination rollout. The coordination structure was initially weak but strengthened over time. Based on the level of coordination activities undertaken, we identified three periods marking different strengths of the coordination mechanisms, these corresponded with different trends in vaccination coverage in the mainland. In the first period (July–December 2021), the coordination mechanism was weak, and vaccine coverage was low, with only 3% of the target population vaccinated on the mainland. In the second period (January–May 2022), when stakeholder engagement was expanded and the coordination mechanism improved, there was a concurrent rise in vaccine coverage from 4% to 25%. In the third period (June–December 2022), coordination was further strengthened, and vaccination strategies were intensified; a corresponding increase in vaccine uptake was observed with coverage reaching 100% of the target population. **Conclusions**: Qualitative insights from the three time periods suggest a positive association between coordination strength and COVID-19 vaccine coverage. Coordination fostered collaboration, enhanced stakeholder engagement, and facilitated data-driven decision making. This enabled Tanzania to overcome complex challenges and achieve significant progress in vaccination coverage. Strong coordination and effective collaboration among stakeholders are essential mechanisms and processes to optimize vaccine delivery resources and ensure the equitable distribution and uptake of vaccines in Tanzania.

## 1. Background

The novel Coronavirus Disease 2019 (COVID-19) was a major public health problem globally [1,2,3]. As of October 2023, the World Health Organization (WHO) had reported over 700 million confirmed cases of COVID-19 and nearly 7 million deaths globally due to the pandemic [4,5]. In the United Republic of Tanzania, as of March 2023, there were approximately 43 thousand cases and 846 deaths attributed to the virus [6,7]. Although no longer a “global health emergency of international concern” [8], COVID-19 remains a global health threat. The deployment and use of COVID-19 vaccines played a pivotal role in arresting the pandemic three years after it was declared [9].

The national rollout of a vaccine is a complex and significant undertaking, made more challenging when the health system is experiencing shock, such as in a pandemic. Vaccines must be rapidly, equitably, and effectively deployed to reach many people in a pandemic. The success of introducing a new vaccine hinges on the convergence of several critical factors, including a thorough understanding of the epidemiologic need and the vaccine’s effectiveness; strong political commitment; vaccine and funding availability; readiness of immunization systems (cold chain, human resources, program management); and acceptance of the vaccine by the target population [10,11].

In many low- and middle-income countries (LMICs), COVID-19 vaccine deployment posed a multifaceted challenge to national immunization programs ranging from initial vaccine scarcity to vaccine hesitancy [12] and vaccine delivery challenges [13,14]. Furthermore, COVID-19 vaccines, which were targeted at the widest age group in any country’s immunization program, including adults, special groups like the elderly, individuals with comorbidities, and pregnant women [15,16], came with unique challenges. With many countries lacking established adult vaccination platforms, the vaccine introduction placed additional demands on weak health systems already strained by the pandemic [17,18,19].

Despite these challenges, some country programs were able to make good progress in achieving their vaccination coverage targets. Tanzania is one of such countries. The country went from effectively mitigating initial political hesitancy surrounding COVID-19 and the vaccines [20,21,22] to securing a stable vaccine supply, optimizing service delivery, and implementing digital solutions for registration and data management. By March 2023, approximately 40 million doses of COVID-19 vaccines had been administered in the country, representing a fully vaccinated coverage of 52.5% [23], based on the total population. Notably, this success was achieved in the context of a multi-partner effort that required coordinated efforts.

Political buy-in, cross-sectoral collaboration, and strong coordination have been credited with the achievement in COVID-19 vaccination rates in Tanzania [24]. Coordination is one of the six pillars of the immunization system building blocks. It refers to the process of organizing and aligning various elements, activities, or individuals to work together harmoniously and effectively toward a common goal or objective. It is one of the first pillars of the World Health Organization’s COVID-19 Strategic Preparedness and Response plan, a monitoring and evaluation framework [25]. Coordination mechanisms that leverage partners’ expertise and foster collaborative dialog [25] are critical strategies for successful emergency management and COVID-19 vaccination rollout. These mechanisms are effective because they help with planning and financing and ensure accountability and transparency in monitoring progress and identifying gaps [26].

Additionally, intersectoral partnerships serve as a vital strategy for addressing crises and routine developmental challenges in developing countries [27]. Coordinated and well-managed partnerships, as well as timely planning, swift execution, community engagement, and multi-sectoral collaboration, are essential for successful initiatives like Africa’s COVID-19 vaccination rollout. Financial support and fostering demand for integrated, life-course vaccination approaches are also critical to achieving sustainable health intervention [28,29].

Gooding et al. [30] provide a useful framework for understanding the key elements that enable effective coordination in health systems during emergencies. These principles—prioritizing inclusivity, establishing appropriate structures, ensuring necessary capacity, and leveraging political leadership—offer a foundation for analyzing the coordination processes involved in the Tanzania COVID-19 vaccine rollout.

Building on this framework, our paper examines how the COVID-19 vaccine rollout in Tanzania was coordinated, focusing on the coordination structure, partnerships involved, and strategies employed over time, and how these correlate with vaccine coverage temporally. By comparing changes in coordination practices over time with trends in vaccination coverage, this analysis seeks to uncover potential relationships between coordination strength and program effectiveness. While not a statistical analysis, this ecological approach offers valuable insights into the role of coordination in vaccine deployment, providing lessons that may apply to other LMICs facing similar challenges in pandemic preparedness and vaccine introduction.

## 2. Methods

### 2.1. Study Setting

This work was conducted in the United Republic of Tanzania, a union between Tanzania Mainland and Zanzibar Island. See Appendix A. It is the largest country in East Africa occupying a surface area of about 945,087 km^2^ with a current population of 61,741,120, as reported in the 2022 census [31]. Tanzania’s mainland is divided into 26 regions and 184 councils. Zanzibar is made of two main islands (Unguja and Pemba) and other smaller islands, with 11 districts in five regions. The Ministry of Health in Tanzania Mainland and Zanzibar regulates, supports, and coordinates the functions of the health sector and all its interventions including the rollout of COVID-19 vaccination. As in other African countries, childhood immunization and COVID-19 services are provided countrywide and are free of charge in public health facilities and in some selected private health facilities. The immunization program is domiciled within the Immunization and Vaccination Department (IVD) of the Ministry of Health, which provides technical guidance and oversight. The regional and district councils are responsible for delivering health services, including immunization services within their areas of jurisdiction. They report administratively to the President’s Office of Regional Administration and Local Government (PORALG). Concurrently, the health sector is also supported by development partners, the private sector, Faith-Based Organizations (FBOs), and non-governmental organizations (NGOs) in ensuring access to quality health services for all.

### 2.2. Study Design and Data Source

This was a mixed analysis approach that triangulated qualitative findings from three sources—a traditional literature review, a desk review, and two stakeholder workshops—with a quantitative analysis of COVID-19 vaccination coverage [32]. The literature review was carried out on Pubmed and Google Scholar to help define the components of the qualitative analytic framework. The desk review examined available vaccination policy documents and guidelines, the national COVID-19 vaccination deployment plan, country-level meeting and coordination reports, COVID-19 national vaccination reports, press releases, and acceleration plans for Tanzania Mainland and Zanzibar. Data on the coordination mechanisms and processes were extracted from the relevant documents. Consultations were conducted during two documentation workshops with teams from the national, regional, and district MOH and PORALG; representatives from the USAID-funded Momentum Country Global Leadership program, comprising researchers from Johns Hopkins International Vaccine Access Center (IVAC) and Jhpiego; and researchers from Muhimbili University College and Health and Allied Science (MUHAS), Tanzania. This workshop provided the space for stakeholders to iterate on the analytic framework and synthesize and contextualize the program coordination information.

For the quantitative analysis, data on the number of people vaccinated and the target population for vaccination were obtained from the CHANJOCOVID system, a COVID-specific instance hosted on the Tanzania District Health Information System (DHIS2) [33]. This system facilitated the data collection, reporting, and monitoring of COVID-19 vaccination services in Tanzania. Data access is granted by the Government of Tanzania. Data quality checks were conducted regularly by the MOH to ensure the accuracy of the DHIS2 data.

### 2.3. Analysis

The writing team first conducted a comprehensive review of the gathered documents. They abstracted information related to the coordination mechanisms. They categorized them into three distinct analytic domains: the architecture of the COVID-19 vaccination coordination structure; the roles, and responsibilities of members of the coordination committees; and the processes involved in coordination, as well as the actions of policymakers, program planners, and service providers at both the national and subnational levels. These were analyzed with a chronological lens to describe the strength of the coordination mechanisms over time. The team also extracted insights on lessons learned and best practices.

The second step of the analysis was to produce time trends in the COVID-19 vaccine coverage. Vaccine coverage was calculated as the number of fully vaccinated persons divided by the target population, defined as those aged 18 years and above. The analysis resulted in vaccine coverage rates above 100% likely due to under-estimated denominators, a common challenge with administrative immunization data. To address this, whenever coverage was above 100%, it was truncated at 100%. We also stratified the analysis by age category (18–60 years and above 60 years), gender, geographical region (all 31 regions in both Tanzania Mainland and Zanzibar), and vaccine type. The descriptive analysis was carried out using Microsoft Excel.

The third step of the analysis involved qualitatively examining the relationship between the strength of coordination mechanisms and observed coverage trends. This included triangulating qualitative insights on the effectiveness of coordination mechanisms during different time periods to infer how coordination strength and program implementation intensity influenced COVID-19 vaccine coverage trends. Additionally, the team identified lessons learned and best practices to inform future efforts.

### 2.4. Ethical Consideration

Ethical approval was not sought for this study as data were obtained from secondary sources, i.e., program documents, published papers, and the CHANJOCOVID system. Data from the CHANJOCOVID system were aggregate, non-identifiable data.

## 3. Results

### 3.1. Architecture of COVID-19 Vaccination Coordination Meachanism in Mainland and Zanzibar

In response to the COVID-19 outbreak, three high-level coordination committees were established at the national level to guide the national response. The first and second of these committees operated at the policy level. They include the National Taskforce (NTF), led by the Prime Minister, and the Inter-Ministerial Committee (IMC), led by the Chief Secretary. The third high-level committee, which coordinated all technical guidance, was the Technical Task Committee (TTC) led by the Permanent Secretary of the Ministry of Health. Operationally, the NTF held the overarching responsibility for national coordination, oversaw the multi-sectoral response, and was run from the Prime Minister’s office. Similarly, for the TTC, the Chief Medical Officer (CMO), who was also the Incident Manager, was designated to chair all technical meetings. He was the overall coordinator of the country’s response to the pandemic through the Incident Management System, which was tasked with ensuring the country contained the COVID-19 outbreak while maintaining essential health services.

**Figure 1:**
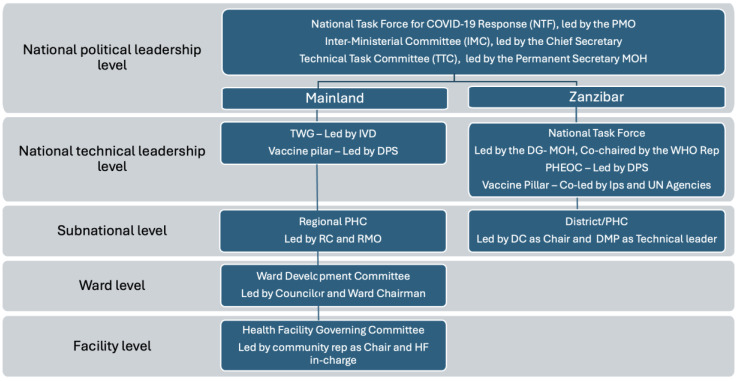
The architecture of the coordination mechanism for the COVID-19 vaccination rollout in Tanzania Mainland and Zanzibar.

The COVID-19 vaccination coordination structure sat under the NTF. The Ministry of Health established multiple committees to coordinate COVID-19 vaccination response in both the Mainland and Zanzibar. The same coordination mechanism was designed at the subnational level building on existing national and subnational coordination and leadership structures. For a detailed architecture of the coordination mechanism, see Figure 1.

Different stakeholder groups were part of the coordinating structure for the COVID-19 vaccine rollout at national and subnational levels. Specifically, the technical working groups and vaccine pillars included implementing partners, and several non-governmental and civil society organizations that supported the government’s efforts in scaling up COVID-19 vaccination in the country. This strategy facilitated resource mobilization and the involvement of national leaders and stakeholders to address the pandemic.

### 3.2. Roles and Responsibilities of the Committees Within the COVID-19 Vaccination Coordination Architecture

The COVID-19 rollout processes were supported by multiple stakeholders at national and subnational levels including both governmental structures and non-governmental organizations (NGOs). The roles and responsibilities at each level are described in the National Guideline for COVID-19 Vaccination. The Ministry of Health, through the national-level coordination committee, specifically the IVD-TWG, developed policy guidelines and technical tools to guide and support efficient coordination and effective implementation to deliver standardized COVID-19 vaccination services countrywide. The specific roles and responsibilities of the stakeholders involved in execution from the national to subnational level in Tanzania Mainland and Zanzibar are presented in Table 1.

### 3.3. Processes Involved in COVID-19 Vaccination Coordination

The Ministry of Health (MOH), through the IVD and the various coordination committees at the national level, including the Multi-sectoral National Task Force (NTF), technical working group, and COVID-19 vaccine pillar, played a central role in the management of the COVID-19 vaccination response, providing strategic leadership, overseeing activities, and supervising the response effort.

To ensure the effective management of the COVID-19 vaccination efforts, the MOH, through the IVD, offered technical guidance to the Regional and Council Health Management Teams (R/CHMTs) and implementing partners (IPs) in all 31 regions. In response to the pandemic, the MOH also strengthened and supported regional and district coordination committees in all 31 regions. This included offering COVID-19 vaccination services in over 7500 facilities and other immunization sites. MOH/IVD pulled in donors and implementing partners to support improving access to COVID-19 vaccination countrywide.

Monthly coordination meetings by members of the National Task Force as well as meetings of the primary healthcare committee (PHC) contributed to keeping the multiple key stakeholders engaged with the planning and implementation of the COVID-19 vaccination interventions. PHC meetings were organized at the regional and district levels. They are existing structures under PORALG led by the Regional Commissioner and District Commissioner at their respective levels. These meetings also provided an opportunity for learning from each other. Furthermore, through weekly vaccine pillar meetings, the COVID-19 vaccination status was discussed with all key stakeholders. Regional data were analyzed to assess performance, comparing performance across regions. Based on the data analysis, strategies were developed to address the identified gaps, to drive program improvements.

The MOH/IVD, through the TWG and vaccine pillar, facilitated and coordinated the training of trainers and service providers in all 31 regions. To better understand the situation in the field, joint supportive supervision visits were organized by the MOH to assess program performance and support providers to implement according to the set standards. Three expanded coordination meetings were conducted in May and June 2022 that involved MoH, PORALG, RHMT, and partners (WHO, UNICEF, USAID, CDC, and DOD) and all regional implementing partners to facilitate partners’ mapping, target setting, and microplanning aligned to regional and council-level microplans.

### 3.4. Trends in COVID-19 Vaccination Outcomes in Tanzania

**Figure 2:**
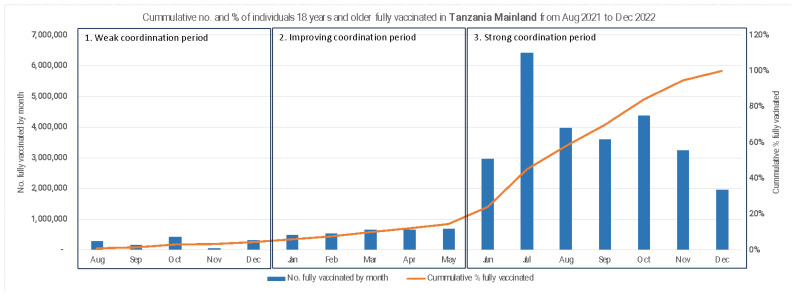
Cumulative number and percent of individuals 18 years and older fully vaccinated in Tanzania Mainland from August 2021 to December 2022.

COVID-19 vaccination was launched in July 2021 in Tanzania after the country secured millions of vaccine doses from the COVAX facility. The vaccine was deployed simultaneously in all 31 regions of the Republic. By December 2021, five months after the vaccine launch, cumulative COVID-19 vaccine coverage changed little, rising only from 1% to 3% in the mainland. The first half of 2022 saw cumulative vaccine coverage increase slowly, by less than 2% per month. An inflection point was reached in June 2022, after which vaccine coverage rose rapidly at almost 14% per month until it hit 100% in December 2022 (Figure 2). The COVID-19 vaccination coverage varied across regions in the mainland from 87% in Morogoro to 100% in Kilimanjaro. Among the fully vaccinated population, 54.4% were women and 45.6% were men.

**Figure 3:**
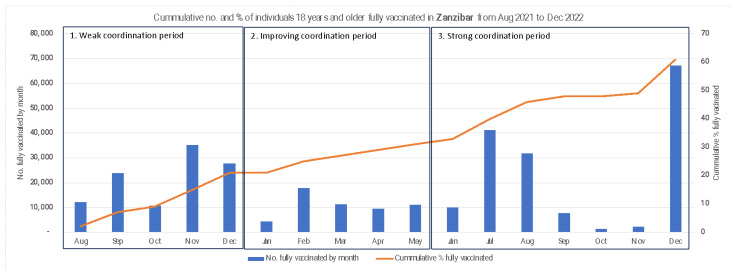
Cumulative number and percent of individuals 18 years and older fully vaccinated in Zanzibar from August 2021 to December 2022.

In Zanzibar, the cumulative coverage trends show a uniform increase over the same period, rising from 2% in August 2021 to peak at 61% in December 2022. Monthly vaccination numbers, however, show a tri-modal spike in November and December 2021, July and August 2022, and December 2022 (Figure 3).

By the end of 2022, sixteen months after the COVID-19 vaccine was introduced, a total of 31,185,231 people were fully vaccinated in the mainland, and 326,537 were fully vaccinated in Zanzibar.

Five types of COVID-19 vaccines were deployed in Tanzania (vaccines by Johnson & Johnson, Moderna, Pfizer, and the Sinovac and Sinopharm products). The single-dose Johnson and Johnson vaccine accounted for the highest proportion of fully vaccinated people. Of the fully vaccinated individuals in the mainland and Zanzibar, 84.3% and 81.1%, respectively, had received the J&J vaccine. Of those vaccinated in Zanzibar, 12% were over 60 years old. This compares favorably with the demographic profile of the country, where about 12% of the adult population is above 60 years of age.

### 3.5. Observed Pattern Between Coordination Strength and COVID-19 Vaccine Coverage

In this section, we describe how the coordination mechanism evolved over the period of vaccine rollout, logically connecting the evolution of the structure and function of the coordinating mechanism, with outcomes of COVID-19 vaccine coverage at different time periods. From its inception in July 2021 to its maturity by December 2022, our analysis identified three distinct time periods with regard to the coordination mechanism. These periods also correspond to three observed time trends in the cumulative coverage rates reported on the mainland.

### 3.6. Weak Coordination Period 1: July to December 2021

In the initial coordination period, spanning the first five months of vaccine deployment from August to December 2021, coordination structure and power were centralized within national governmental entities, namely the Ministry of Health (MOH) and the President’s Office for Regional Administration and Local Government (PORALG). Although immunization donors and implementing partners operated within the country, their efforts were disjointed due in part to the inadequate inclusivity of the existing government-led coordination architecture in Tanzania. This lack of cohesion resulted in disparate planning, implementation, reporting, and communication efforts. With different actors pursuing their operational agendas, collective action was weak, and vaccination efforts were inefficient and less effective. During this period, there was a notable absence of clear guidance on stakeholder involvement and a lack of an accountability framework to monitor the support provided by program partners at regional and other subnational levels where implementation occurred. Additionally, the rollout effort suffered from under-resourcing, impeding the effective scaling up of activities. Despite government initiatives to enhance service delivery—such as strengthening facilities to offer COVID-19 vaccination services and conducting sensitizations in all 31 regions to support the mobilization and delivery of vaccination services—only 3% of the target population in the mainland and 20.6% in Zanzibar were fully vaccinated by the end of December 2021. This phase was therefore characterized by sluggish COVID-19 vaccination uptake and a need for intensified programmatic actions on a broader scale.

### 3.7. Improving Coordination Period 2: January to May 2022

Recognizing the need to involve more stakeholders in the rollout effort, the Government of Tanzania, through the MOH and PORALG, began to revamp the coordination structure to accelerate the uptake of COVID-19 vaccination. This period roughly corresponds to the time between January and June 2022. During this period, the government expanded its engagement at all levels, bringing in diverse national and subnational actors into the technical working groups, including policymakers, donors, implementing partners, public health practitioners, religious leaders, community leaders, influential people, CSOs, and other non-state actors to harness their expertise, resources, social assets, and financial resources to enhance access and coverage of COVID-19 vaccination in all 31 regions of Tanzania. In addition to structural changes, functional changes also occurred. The MOH took proactive steps to improve the coordination, planning, implementation, and communication of COVID-19 vaccination efforts. The MOH and PORALG wrote letters to all regional and district commissioners, to task their medical officers to develop COVID vaccination implementation plans and report progress on implementation regularly. A partner mapping was conducted to identify where various partners were working and what activities donors were funding. The mapping also showed where resources could be aggregated and optimized to implement critical activities—including health worker training, supportive supervision, and logistics for vaccines and vaccinators. The partner mapping helped to delineate specific roles and areas of operation to prevent the duplication of efforts and ensure that all 31 regions in Tanzania received the necessary support. This resulted in each region having at least one implementing partner (IP) to support demand creation, service delivery, and the management of the cold chain system.

Subnational political leaders and influencers were encouraged to express their support for vaccination publicly. Many political leaders received their vaccinations in public gatherings and dispelled myths about the vaccine. It was important to demonstrate political support for the vaccination and signal a departure from the government’s previous skeptical stance on the COVID-19 vaccine to boost public confidence.

Principles that underscored the coordination and implementation approaches were transparency, government leadership, shared responsibility, joint planning, strong communication, and responsive feedback. The technical working group was the central point for coordination and problem solving. Data were used effectively, and donors allowed their implementing partners some flexibility to leverage current activities and funding and adjust their programmatic boundaries to allow for responsive action. During this period, vaccine coverage rose from 4% to 25%.

### 3.8. Strong Coordination Period 3: June to December 2022

The evolution of the coordination mechanism began in July 2022, after the structural and functional changes had been well underway, and the results of a more effective coordination mechanism were beginning to manifest. During this period, there was a steep rise in the number of people receiving the COVID-19 vaccine in Tanzania Mainland, as shown in Figure 2. Deployment activities during this period built on progress made in prior months; for example, by June 2022, large numbers of health workers had been trained to offer COVID-19 vaccination services, which made it easy to deploy them at scale for vaccination service delivery. In addition, heightened political commitment and strengthened coordination at the national, regional, and district levels, enabled high-impact vaccination strategies to be scaled up efficiently across the 31 regions. For example, the vaccination strategies were expanded beyond the fixed and outreach services, to include house-to-house vaccination. This markedly improved COVID-19 vaccine access and uptake. This intensified vaccination campaign led by implementing partners in collaboration with Regional and Council Health Management Teams (RHMTs and CHMTs) was supported with strategic community engagement through targeted Information, Education, and Communication (IEC) activities by trusted and influential messengers. This was instrumental in countering COVID-19 vaccine misinformation and rumors to improve vaccine acceptance.

Collaborating with partners, the government secured most vaccines, including those requiring multiple doses for optimal immunogenicity. Implementing partners also provided logistics support to increase the availability of vaccines across all immunization sites. This allowed for the expansion of services and the implementation of targeted strategies to reach priority groups for COVID-19 vaccination. Civil society organizations and local agencies were actively engaged and coordinated at the subnational level to facilitate community sensitization efforts during mass COVID-19 vaccination campaigns. This coordination with various actors resulted in heightened community awareness regarding the availability of COVID-19 vaccination services and expanded opportunities for hard-to-reach populations to access these services.

The continuous political support and coordinated approach were essential in ensuring accountability among key players, in contrast to situations where different stakeholders operated in isolation. With strong coordination in place, monitoring tools and the CHANJOCOVID system were developed. Facility-based vaccination registers, tally sheets, and client cards were extensively produced and distributed with the support of donors and implementing partners to ensure the proper and standardized documentation of records at all levels, both in the mainland and Zanzibar.

To foster accountability and an effective response, key stakeholders were held responsible for fulfilling their duties through weekly vaccine pillar meetings at the national level and primary healthcare committee meetings at the subnational levels. Periodic reviews to compare regional and district performances with set targets were conducted during coordination meetings of regional and council primary healthcare committees, overseen by Regional and District Commissioners. These regular assessments identified underperforming regions and councils, prompting the allocation of additional support to enhance COVID-19 vaccination uptake. Harmonized stakeholder efforts, along with timely data-driven decision making enhanced the operational capacity that led to achieving and surpassing the national goal of 70% vaccination rate among the eligible population by December 2022.

## 4. Discussion

In 2023, the WHO reported Tanzania as one of the best-performing countries on COVID-19 vaccine coverage among 34 African countries supported by the COVID-19 Vaccine Delivery Partnership [24]. In this paper, we aimed to explore what contributed to Tanzania’s success by examining how coordination may have played a role in the successful rollout of the vaccine given that coordination is an essential pillar of the immunization system’s building blocks. We found that the Government of Tanzania established and strengthened the coordination mechanisms for the COVID-19 vaccine between July 2021 and December 2022, building on existing structures at the national and subnational levels.

In examining the architecture, roles, processes, and activities of the vaccination coordination mechanism, we identified three high-level committees set up by the government —the National Taskforce (NTF), Inter-Ministerial Committee (IMC), and Technical Task Committee (TTC)—which were central to managing the national response to COVID-19. The TTC provided technical guidance for the COVID-19 vaccine rollout at the national level. Similarly, coordination mechanisms extended sub nationally, leveraging Regional and Council Health Management Teams (R/CHMTs) to ensure alignment with national priorities and local implementation. We found that the composition and activities of the coordination mechanisms became stronger over time, and the improved structure and functionality of the committees were instrumental to the success of the vaccine deployment effort. The strengthened coordination structure was not only multi-level (operating at both the national and subnational levels), it was also multi-partner (comprising government officials, technical experts, donors, implementing partners, civil society, community leaders, etc.), and it was integrated. The coordinating committees were designed to provide strategic direction, oversight, and guidance throughout the vaccination rollout. Through convening and influencing stakeholders at various levels the government was able to rally support for vaccine deployment efforts toward meeting national vaccination targets [34].

In many countries, the COVID-19 pandemic was not just a public health problem, but also a challenge with broad cultural, socio-economic, cultural, and political components [35]. Therefore, the response was seen as a whole-of-society effort, which could not be managed by the government alone. Similarly, for COVID-19 vaccine deployment in Tanzania, it was essential to involve a diversity of actors to support different aspects of the rollout given the multifaceted challenges associated with the vaccine deployment [23]. The involvement of diverse stakeholders, including governmental bodies, NGOs, and international agencies, was important. Experience from Iran corroborates our findings that the coordination and engagement of key stakeholders facilitate the effective management of COVID-19 vaccination services [36]. By leveraging the expertise, knowledge, social ties community influence, and monetary and material resources of different stakeholders, the government was able to address the multidimensional need to fix supply chain issues, implement targeted communication strategies to combat vaccine hesitancy, and build public trust and acceptance of the vaccine while scaling up service delivery strategies. The effective coordination of the efforts of the multi-sectoral partnership was key to harnessing the strengths and contributions of the various partners [23,34].

A critical aspect of this coordination was having clear documented terms of reference of committee members to ensure clarity of roles and responsibilities and efficient decision making. Similarly, COVID-19 control efforts in Jordan were initiated by establishing a multi-sectoral COVID-19 vaccination National Coordinating Committee [24]. The early realization of partners’ involvement led to successful control efforts and initial planning that involved multi-sectoral partners including, the private sector, which fostered accessible and equitable vaccine distribution in these settings [36,37]. Effective strategies for administration, coordination, and vaccine distribution were also reported in Cuba, which resulted in significantly improved COVID-19 vaccination uptake [38]. In contrast, inefficient coordination systems have been mentioned as one of the most important challenges facing vaccination systems in low- and middle-income countries [39,40]. Similarly, a study in the US suggests that the poor integration of network governance into the hierarchical governance structure leads to weak crisis coordination [41].

Our analysis also shows that it may take time for coordination mechanisms to become strong enough to become efficient and impactful. In line with the country ownership guiding principle of the Immunization Agenda 2030 [42], the government must be in the driver’s seat and be willing to bring on board other partners early enough in the process. This did not happen in the first few months after the COVID-19 vaccine’s introduction in Tanzania. From July to December 2021, coordination efforts were fragmented, resulting in the poor implementation of vaccine delivery efforts, ineffective demand generation activities, and slow uptake of vaccines. However, recognizing the need for enhanced coordination, the government revamped the structure, bringing in community-level structures and engaging a broader array of stakeholders. This shift toward a more inclusive and collaborative approach led to significant improvements in vaccine coverage rates [23]. Early and effective coordination may cut down the ramp-up period by bringing a surge of resources that work together simultaneously to improve the efficiency and effectiveness of planning, capacity building, supplies and materials, implementation, monitoring, and learning [34].

Another important insight from our study is the critical role of regular monitoring and evaluation to support data-driven decision making in shaping vaccination strategies. Using information from the CHANJOCOVID database system and other monitoring tools, stakeholders were able to track progress, identify bottlenecks, and tailor interventions accordingly. This iterative process of learning and adaptation was instrumental in driving continuous improvement in vaccination coverage.

A key limitation of this study is its reliance on descriptive analysis and narrative synthesis, which restricts the ability to draw causal inference. The observed trends in COVID-19 vaccination coverage may be influenced by a range of unmeasured contextual or programmatic factors. We infer a relationship between coordination strength and vaccination uptake on the mainland, but this association was not evident in Zanzibar, where increases in coverage were observed even during the periods of weak coordination. This suggests that coordination may not hold the same explanatory value across the two contexts. The underlying reasons for this divergence remain unclear and warrant further investigation of the hypothesized association between coordination and coverage using other methods that would allow for formal statistical analysis.

Another limitation of our paper is the challenge with data quality as we observed coverage rates above 100%. This may be from faulty denominators, the result of inaccurate projections from outdated census figures, a common problem in some LMICs [43]. There remains a need to improve campaigns and routine immunization data systems to provide accurate numerators and denominators for effective program monitoring and action.

Despite the successes achieved, our study also highlights ongoing challenges and areas for improvement. For instance, while vaccine coverage was high overall, disparities across regions were observed. Addressing these inequities will require targeted interventions, including tailored communication strategies, community outreach efforts, and strengthened healthcare delivery systems in underserved areas.

Given the transient nature of the pandemic response, future research should examine the dynamics of coordination within the context of transitioning from an emergency response to a more routine public health approach. This could provide insights into improving vaccination efforts and ensuring preparedness for future public health challenges.

Moving forward, the following are implications for managers:

Clear delineation and action of government leadership roles are essential during new vaccine deployment to address public health crises. Managers should prioritize the establishment of overarching governance and accountability mechanisms to ensure effective coordination and response efforts. Building on existing coordination frameworks at both national and subnational levels can help expedite response actions. Leveraging these established systems allows for more efficient planning and execution of the necessary interventions.

Involving a diverse range of stakeholders, including technical groups and civil society organizations, plays a critical role in resource mobilization and fostering public engagement. Managers should prioritize multi-sectoral collaboration to optimize resource use and build public trust. Effective inclusion of stakeholders strengthens the overall response and ensures that diverse perspectives and expertise contribute to problem-solving. Furthermore, strengthening local capacity through training and leveraging community influencers to counter vaccine misinformation is important. Managers should invest in community-oriented strategies to address barriers to service uptake.

Regular data reviews and performance comparisons across regions helped identify gaps and adapt strategies. Managers should establish robust data monitoring systems to inform responsive decision making.

## 5. Conclusions

In conclusion, our findings underscore the critical role of coordination in facilitating the successful deployment of COVID-19 vaccines. By fostering collaboration, enhancing stakeholder engagement, and prioritizing data-driven decision making, Tanzania was able to overcome complex challenges and achieve significant progress in vaccination coverage. These lessons can be used by other countries to improve their vaccination programs and pandemic preparedness.

## Figures and Tables

**Table 1:** Coordination roles and responsibilities of stakeholders in the national and subnational COVID-19 vaccine coordination structure.

Multi-sectoral National Task Force (NTF)	Overall national coordination and leadership of the multi-sectoral response
Technical Working Group	Providing recommendations to the ICC on all matters about improving immunization services as well as introducing the new vaccine Setting standards and developing guidelines for vaccination microplanning and implementation Advocacy and resource mobilization Conducting training and supporting the development of microplans Ensuring the presence of adequate cold chain and logistics arrangements for vaccine storage, distribution, and administration to the target population Mapping and distribution of implementing partners at a subnational level
COVID 19 Vaccine Pillar	Providing technical recommendations to key stakeholders, donors, and implementing partners to support scaling up COVID-19 vaccination services Providing technical advice to implementing partners for effective COVID-19 vaccination programming Developing strategies for COVID-19 vaccination performance improvement Setting standards for rolling out COVID-19 vaccination implementation guidelines and microplanning
Regional and District PHC	Development of macro/microplans and plan of action for the region/district Coordination and implementation of training, microplanning, supervision, and monitoring Analysis and synthesis of microplans from Councils to guide appropriate strategies Distribution of COVID-19 vaccines and other supplies including IPC supplies Social mobilization and communication to generate demands
Ward Development Committee	Monitoring implementation of COVID-19 vaccination at ward level Monitoring COVID-19 vaccine uptake Facilitate community mobilization activities for COVID-19 vaccination to improve coverage
Implementing partners (Non-Governmental Organizations)	Support scaling up and facilitate the implementation of COVID-19 vaccination activities Financial support for COVID-19 vaccination campaigns and activities

## Data Availability

The original contributions presented in the study are included in the article. Further inquiries can be directed to the corresponding author.
